# Unmasking the ancestral activity of integron integrases reveals a smooth evolutionary transition during functional innovation

**DOI:** 10.1038/ncomms10937

**Published:** 2016-03-10

**Authors:** Jose Antonio Escudero, Celine Loot, Vincent Parissi, Aleksandra Nivina, Christiane Bouchier, Didier Mazel

**Affiliations:** 1 Institut Pasteur, Unité de Plasticité du Génome Bactérien, Département Génomes et Génétique, 28 Rue du Dr Roux, 75015 Paris, France; 2 CNRS, UMR3525, 28 Rue du Dr Roux, 75015 Paris, France; 3 Laboratoire de Microbiologie Fondamentale et Pathogénicité UMR-CNRS 5234. Bat 3A 3ème étage, 146 rue Léo Saignat, 33076 Bordeaux, France; 4 Université Paris Descartes, Sorbonne Paris Cité, 75006 Paris, France; 5 Institut Pasteur, Genomic Platform, 28 Rue du Dr Roux, 75015 Paris, France

## Abstract

Tyrosine (Y)-recombinases have evolved to deliver mechanistically different reactions on a variety of substrates, but these evolutionary transitions are poorly understood. Among them, integron integrases are hybrid systems recombining single- and double-stranded DNA partners. These reactions are asymmetric and need a replicative resolution pathway, an exception to the canonical second strand exchange model of Y-recombinases. Integron integrases possess a specific domain for this specialized pathway. Here we show that despite this, integrases are still capable of efficiently operating the ancestral second strand exchange in symmetrical reactions between double-stranded substrates. During these reactions, both strands are reactive and Holliday junction resolution can follow either pathway. A novel deep-sequencing approach allows mapping of the crossover point for the second strand exchange. The persistence of the ancestral activity in integrases illustrates their robustness and shows that innovation towards new recombination substrates and resolution pathways was a smooth evolutionary process.

## Abstract (web summary)

The integron integrases have evolved to perform recombination of single and double stranded DNA. Here the authors show that the ancestral pathway is still functional at double stranded sites, revealing the evolution towards the modern resolution pathway.

## Graphical abstract

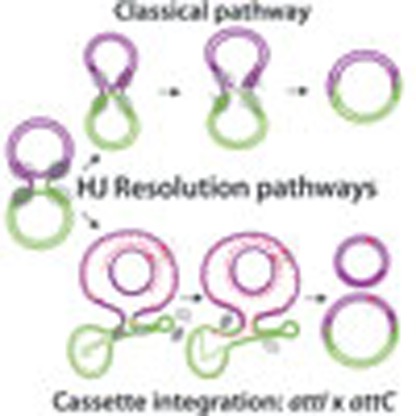

Tyrosine (Y)-recombinases mediate recombination between two specific DNA sites to deliver a variety of biological functions (for a review see (ref. 1)). They are present in the three domains of life but are most widespread among prokaryotes, where they play fundamental roles such as the integration/excision of viral genomes^2^, the alternation of gene expression^3^ and the resolution of deleterious chromosome dimers arising during replication^4^. Members of this family show a conserved fold of the catalytic domain including a three-dimensional clustering of highly conserved RKHRH residues. Y-recombinases typically recognize specific DNA sites comprising two inverted binding domains separated by a 6–8 bp spacer. The recombination reaction is a stepwise process that starts with the cleavage and transfer of one strand, leading to the transient formation of a Holliday junction (HJ) that is further resolved through a second exchange of strands. This sequence of steps, which we will refer to as the classical pathway (Fig. 1a), is highly conserved among Y-recombinases. Type IB topoisomerases and telomere resolvases share the fold and catalytic chemistry, and can therefore be considered as distant relatives within the same superfamily, despite sequence divergence^1^,^5^,^6^,^7^. This implies that recombinases have evolved to deliver mechanistically distinct reactions (for example, the cleavage of only one strand by topoisomerases), and to recognize structurally different substrates (for example, hairpins in telomeres). Such evolvability is likely facilitated by the robustness of the fold^6^ (its capacity to accommodate mutations while preserving its tertiary structure), as well as the modularity of protein function through the combination of different functional domains. Nevertheless, information on evolutionary pathways that enable functional innovation among these enzymes is scarce.

Integrons are recombination platforms that allow bacteria to evolve and adapt rapidly through the acquisition, stockpiling and reordering of genes embedded in mobile genetic elements named integron cassettes^8^,^9^,^10^ (Fig. 1b). They are ancient structures that have driven bacterial evolution for hundreds of millions of years^11^, and, through their mobilization onto conjugative plasmids, they have played a critical role in the rise of multidrug resistance among clinically relevant bacteria^12^. Recombination in integrons is governed by integron integrases^13^ (hereafter integrases), a distinct group of Y-recombinases closely related to XerCD recombinases^8^. Integrases show the conserved fold of the family, as well as the RKHRH pentad of residues in the active site (Fig. 1c). Incorporation of cassettes into the integron platform occurs through site-specific recombination between the *attC* site in the cassette and the *attI* site in the integron^14^. Genes in cassettes are then expressed from the Pc promoter within the integron platform (Fig. 1b). Cassette recombination represents an exception to the archetypical reaction mediated by Y-recombinases, as a consequence of the structural peculiarities of *attC* sites. These sites show no sequence conservation, except for the crossover point on the bottom strand (bs) (5′- AAC -3′), that provides microhomology with *attI* sites, necessary for strand transfer^15^. Instead, *attC* sites share a conserved palindromic structure, and are only recognized as a folded single strand (ss)^16^ (Fig. 1d). Recombination between a ss-*attC* and the double strand (ds)-*attI* site forms an asymmetric HJ (aHJ) (Fig. 1a) that cannot be resolved through a second exchange of strands since it would linearize the replicon and be abortive^17^. During *attC* site folding, the imperfect pairing of both arms of the bs allows for the protrusion of a set of extrahelical bases (EHBs) (Fig. 1d) that are key in strand selectivity, favouring the recombination of the bs^18^,^19^. EHBs also induce conformational changes in one integrase monomer during the reaction, pulling apart the tyrosine residue and avoiding the nucleophilic attack on the L box of the *attC* site^20^ (Fig. 1d). This prevents the second strand exchange, and the aHJ is then resolved through replication^21^ (Fig. 1a). To accommodate EHBs, integrases possess a 20-residue-long domain dubbed I2 (ref. 22; Fig. 1c) that has not been identified elsewhere, suggesting that it could have emerged recently^23^ among Y-recombinases. Such domain insertions are well-characterized phenomena in protein evolution^24^, allowing for a modular organization of protein function.

ssDNA is central to the integron, also controlling integrase expression through the SOS response^25^. Integrons are, therefore, unique recombination platforms streamlined to recombine ssDNA through a distinct resolution pathway. They represent a molecular example of functional innovation^26^.

While *attC* sites have been extensively studied^17^,^19^,^20^,^27^,^28^, knowledge is lacking on the recognition and processing of *attI* sites. These are more canonical than *attC* sites, since they are recognized as dsDNA^16^. They have two binding domains, the L and R boxes, although one is degenerate (Fig. 1d), and are generally recognized specifically by their cognate integrases^29^. Some *attI* sites possess accessory binding domains^30^.

Integrase site recognition underscores a remarkable conformational dynamism^31^. Identical monomers recognize ds-*attI* sites by sequence and ss-*attC* sites through structural landmarks. From an evolutionary standpoint, this duality could reflect a single specialized activity (the recombination of ss/ds DNA partners) or two distinct ones: the canonical Y-recombinase activity of integrase ancestors on *attI*, and the functional innovation, that is, the recombination of ssDNA structures, on *attC* sites. For the latter to be true, both classical and innovative activities must be complete: integrases should be capable of resolving HJs through a second strand exchange.

To unmask the ancestral activity of integrases, we have studied the reaction between two *attI* sites. This is a symmetrical reaction involving exclusively ds partners for which resolution through a second strand exchange is theoretically possible, since it is neither abortive nor impeded by EHBs. We show that the top strand of *attI1* is reactive and that HJ resolution can take place through a second strand exchange. We have determined the crossover point through a novel deep-sequencing approach that is applicable to other recombination systems, and validated this approach *in vitro*. Finally, we prove that *attC* sites govern the directionality of the reaction. To our knowledge, integrases are the first example among recombinases of a smooth evolutionary transition between substrates and processing pathways. We discuss the possible origins and the evolutionary implications of the dual activity of integrases, as well as the importance of *attI1* × *attI1* reactions in antibiotic resistance.

## Results

For this work we chose to study *attI1*, the integration site of the most widespread and best-studied integron platform, the Class 1 integron.

### Dispensability of known HJ-processing host factors

The *attI1* site possesses two direct repeats upstream the L–R boxes that make the site ∼65-bp long^30^. It is therefore possible that host factors specialized in homologous recombination, branch migration and resolution of HJ-like structures (such as stalled replication forks), could influence the resolution of the *attI1* × *attI1* HJ, biasing our results. To assess the impact of such host machinery, we have tested the influence of RecA and two well-characterized HJ-processing complexes, RecG^32^ and RuvABC^33^,^34^, using a classic suicide conjugation assay^35^. Recombination rates of mutants of these complexes were not statistically different from those of the wild-type (WT) strain, suggesting that known host HJ-processing systems do not significantly influence the *attI1* × *attI1* reaction (Supplementary Fig. 1).

### Both strands of *attI1* are reactive

In cassette integration reactions, only the bottom strands of *attC* and *attI* sites are exchanged, with the crossover point located within the R boxes of both sites. To test the hypothesis that the *attI1* × *attI1* reaction can take place through both the bottom and the top strand, we used an *attI1* carried on a mismatched covalent circle^21^. These synthetic circles mimic non-replicative plasmids assembled using complementary strands produced separately and hybridized together *in vitro*. This allowed us to specifically tag each strand with a mutation in a restriction site (SacII or NarI) so that the restriction pattern of cointegrates reveals which strand is transferred during recombination (Fig. 2a). In our setting, the transfer and replication of the bottom and the top strands leads, respectively, to a SacII^+^/NarI^−^ and SacII^−^/NarI^+^ restriction pattern. The classical pathway of double-strand exchange would yield cointegrates containing both mismatches that, once replicated, would produce a mixed population of plasmids and hence partial restriction profiles with both enzymes (Fig. 2b). We verified the correct assembly of the DNA circles (as in ref. 21 (Supplementary Fig. 2)) and transformed them into a *mutS*^−^ MG1655 *Escherichia coli* (*E. coli*) strain containing an *attI*-bearing pSU plasmid (p929) as well as a plasmid with the integrase under the control of a P_BAD_ promoter (pBAD-*intI1*). The *mutS*^−^ background ensures that the mismatches are not repaired. By selecting for the circle resistance marker, we recovered clones in which these molecules had recombined with the resident pSU plasmid through an integrase-mediated *attI1* × *attI1* reaction. The mismatch-containing region was PCR amplified from 100 recombinant clones and used for the restriction pattern analysis (Fig. 2c), revealing that both strands of the *attI* site are reactive and can be transferred independently, without a clear bias on strand specificity. This is contrary to what is observed for the *attI1* × *attC* reaction where only the bs of *attI1* is reactive^17^. Some clones showed partial restriction patterns and mixed plasmid populations (Fig. 2d), suggesting the possibility of HJ resolution through second strand exchange. Nevertheless, it could not be ruled out that this pattern was the consequence of two independent recombination reactions involving two different plasmids.

### The integrase resolves HJs through a second strand exchange

To further assess if both strands of the *attI1* site could be transferred during the same reaction, we used a modified version of the *dapA* recombination reporter^36^. In this system, recombination between *attI1* sites leads to reconstitution of the essential gene *dapA*, allowing recombinants to grow on media lacking diaminopimelic acid (DAP, the reticulating agent of peptidoglycan in *E. coli*). Comparison of the number of clones growing with and without DAP yields a recombination frequency for a given reaction. We produced two versions of this reporter system, one with two *attI1* sites in direct orientation, and the other with sites in inverted orientation. Both structures were inserted ectopically in single copy in the chromosome of an *E. coli* MG1655 Δ*dapA* strain. The rationale of this experiment is that, when both sites are in inverted orientation, viable recombinants can only be recovered if HJ resolution takes place through a second strand exchange that inverts the region between both sites. In this orientation the replicative resolution pathway linearizes the chromosome and is hence abortive (Fig. 3a; Supplementary Movies 1 and 2). On the other hand, when sites are in direct orientation both resolution pathways result in the excision of the region between the sites and are hence productive, serving as a control for the experiment. Our results show that integrase expression increased significantly the yield of recombinant clones for sites in direct orientation (44-fold) but also for sites in inverted orientation (72-fold) (Fig. 3b) (verified by PCR). This demonstrates that the *attI1* × *attI1* HJ can be resolved through a second strand exchange, that is, through the classical Y-recombinase pathway. Furthermore, comparison of the increases in recombination frequencies for both orientations proves that the classical resolution pathway is extremely efficient.

To avoid expression of *dapA* before recombination, one *attI1* site was modified 1 bp upstream of the L box to include a stop codon (*attI*_*STOP*_) (Fig. 3c). As a consequence, resolution through branch migration is not possible, suggesting again that host factors were not necessary in the reaction. It implies too that resolution of the HJ had taken place not further than 1 bp upstream of the L box, as confirmed by the change of location of the stop codon after recombination.

A similar experiment was performed using two *attC* sites, *attC*_*aadA7*_ and *attC*_*ereA2*_ (Supplementary Fig. 3). When sites were in direct orientation, we observed an increase in recombination frequencies of five orders of magnitude due to the presence of the integrase. Contrary to our observations for the *attI1* × *attI1* reaction, no clear increase in recombination frequency was observed for *attC* sites in inverted orientation. Sequencing of the few events (*n*=8) in which the central structure was found inverted showed that the site had not been recombined in a double-stranded form. Indeed, the resulting sites were largely conserved, with the presence of moieties of the original sites only visible after the canonical 5′- AAC -3′ cleavage point. Although these inversion events require a second cleavage that could not be elucidated, and therefore seem to be the consequence of illegitimate recombination reactions (possibly between sites on different replicating chromosomes), they are strong proof that *attC* sites are exclusively recombined in their structured form and confirms previous observations^17^.

To further prove that the second strand exchange occurs and is mediated by the integrase, we explored the possibility of impeding the bottom strand recombination. To do so, we mutated the cleavage point in the R box of one *attI1* site in direct and inverted orientation from 5′- AAC -3′ to 5′- AAA -3′ (see *attI*_*AAA*_ in Fig. 3c). It has been observed that a C to A modification drops recombination rates for *attI1* with both *attC* and *attI1* partners^15^,^37^. Our results show a complete abolition of recombination when both sites are in the inverted orientation (that is, when the second strand exchange is needed to resolve the HJ), proving that the bs is no longer reactive. In contrast, recombination rates remain constant when the sites are in direct orientation, proving that integrase-mediated recombination through the ts is still possible for the *attI*_*AAA*_ site, and supporting the previously observed lack of clear bias in strand selection.

### Mapping the ts crossover point using deep sequencing

Recombination between sites on different replicons results in co-integrate molecules bearing two sites. Each of them is composed of two moieties of the initial sites, but when the latter are identical, moieties are not easily identified and the crossover point of the reaction remains cryptic. Such is the case of the *attI1* × *attI1* reaction. Hence, to map the crossover point on the top strand of *attI1*, we built a library of *attI1* sites in which we randomized all base pairs in the L box, the two adjacent base pairs within the spacer region and the C in the crossover point of the R box (Fig. 4a). Using N-containing primers, 10 *attI1*_*N*_ site sets were produced, each bearing random bases in one of the ten positions. These sites were cloned in *pir*-dependent pSW plasmids and mixed to form a small library (*n*=40) of pSW-*attI1*_*N*_ plasmids to be used in our suicide conjugation assay. The rationale of this experiment is that the pattern of segregation of random bases between the two resulting sites in the cointegrate molecule will reveal the precise crossover point in the *attI* top strand (L box). Furthermore, since base composition of the L box can influence IntI1 binding, some *attI* mutants might be poorly recognized. A skew in the proportion of bases in a given position after recombination would underscore the importance of a specific base in the binding process (Fig. 4b). The shortcoming of this experimental set-up is that, as seen before (Fig. 2), the reaction can take place exclusively through the bottom strand (bs-exchange plus replication), without splitting the randomized L box. To overcome this, having confirmed the lack of bs exchange for *attI1*_*AAA*_, we used a pSU-*attI1*_*AAA*_ plasmid as recombination partner for the library, forcing recombination to occur at the randomized L box. Deep sequencing of pSW-*attI1*_*N*_ before recombination confirmed the expected distribution of 7.5% mutated bases in the randomized positions (explained in Methods) (Fig. 4c). Sequencing of the resulting left and right sites in the cointegrate revealed a complementary distribution of random bases among sites, allowing location of the crossover point in the L box between both adenines of the 5′- AAC -3′ triplet (Fig. 4c). It also allowed us to infer the importance of each base for protein–DNA interactions, revealing lower sequence stringency in the central 5′- CT -3′ base pairs of the L box (5′- CCCTAAA -3′), in accordance with previous interference data for the central T base^38^. To confirm the crossover position, we performed the *attI1* × *attI1* reaction *in vitro* using purified MBP-IntI1 protein, which is known to be fully active *in vivo*^39^, and *attI1* fragments bearing a mismatch between the top and the bottom strand in the adenine that is transferred during the reaction (ts sequence 5′- ATC -3′, bs sequence 5′- GTT -3′) (Fig. 5a). Such a mismatch eliminates the microhomology necessary for docking of the transferred strand, and should abolish the transfer of the top strand. While the reaction between two WT *attI1* sites is poorly detected *in vitro*^40^ (likely because HJ intermediates are quickly resolved), the reaction between L-box-mismatched substrates led to a significant accumulation of HJs (Fig. 5b), proving the role of the L-box cleavage in the resolution of these structures, and the dispensability of host factors in the reaction. The complementary effect (that is, the accumulation of HJs due to an R-box mismatch) was not observed, probably because mismatches in the R box led to a significant decrease in integrase binding (Supplementary Fig. 4a). The fact that binding is only affected by R-box mismatches is in accordance with the sequential and cooperative binding of monomers, starting on the R box^38^. As a control, all reactions were performed in parallel with the catalytically inactive version of the integrase, IntI1_Y312F_, for which no transfer activity was observed.

### *attC* sites govern the directionality of the reaction

**Figure 1:**
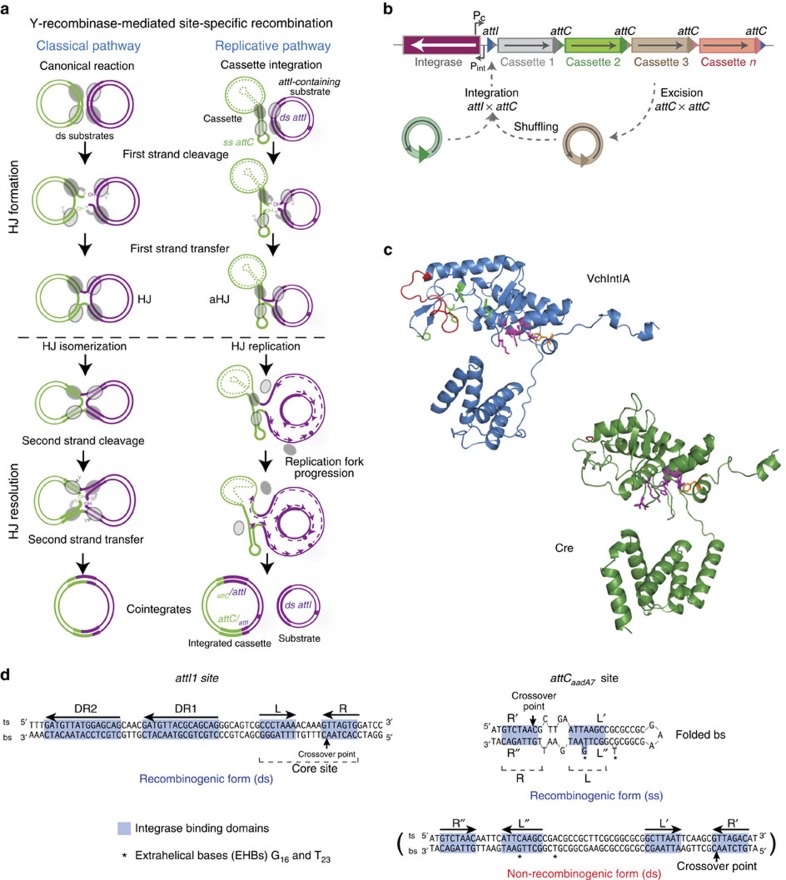
Recombination in the integron. (**a**) Diagram showing the two recombination models among tyrosine recombinases. The left column shows the recombination pathway shared by a majority of Y-recombinases, except the integron integrase for which the pathway is represented in the right column. (**b**) Schematic representation of the integron architecture and recombination reactions. Open reading frames are represented as boxes, with arrowheads showing the direction of transcription. Arrows marked as Pc and Pint represent the promoters of the cassette array and the integrase, respectively. Acquisition of new cassettes involves the recombination of the integron *attI* site and the cassette *attC* site, while cassette excision involves two *attC* sites. Sequential coupling of excision and integration reactions shuffles the order of cassettes within the array. (**c**) Crystal structure of *V. cholerae* integrase^20^ (blue) and Cre^60^ (green). RKHRH residues are depicted as purple sticks. The catalytic tyrosine is shown in orange. Red residues represent the I2 domain in VchIntIA and the residues in Cre that align with those delimiting I2 in VchIntIA. Residues in VchIntIA that interact with EHBs are represented in green. (**d**) Sequence of the Class 1 integron *attI* site (*attI1*), and the *attC*_*aadA7*_ site. aHJ, asymmetric HJ; bs, bottom strand; DR, direct repeats; ds, double stranded; HJ, Holliday junction; ss, single stranded; ts, top strand.

During *attC* site folding, the inverted repeats of the dsDNA molecule hybridize to form the R and L boxes in the hairpin (Fig. 1d). These boxes are present in hairpins arising from the folding of both the top and bottom strands. Despite a clear bias towards bs recombination, top strands are also recombined, although at much lower frequencies. In all cases, the crossover in the *attI* × *attC* reaction takes place in the R boxes of both sites. This means that top-strand recombination integrates cassettes opposite to the Pc promoter and are likely non-functional. Therefore, the similar frequency at which L and R boxes of *attI1* recombine in the *attI1* × *attI1* reaction is in clear opposition to that found for the *attI1* × *attC* reaction. An event that has never been reported is the recombination of *attC* sites through the L box. We sought to detect such recombination events with *attI1*, since they would presumably occur through the L box of *attI1* ts. We reasoned that, since bottom strands of both sites recombine very efficiently through the R boxes, it would be easier to observe such events using the ts of *attC*. Using our conjugation assay, we delivered the ts of *attC*_*aadA7*_ and checked a total of 607 recombinants. Among them, an estimate of 251 had recombined through the ts (the rest having recombined through the resynthesized complementary bs) and only 1 (0.16%) had recombined through the L box with the top strand of *attI1* (Supplementary Fig. 5). Since the EHB T_23_ on the bottom strand impedes the nucleophilic attack on the L box, we assessed if the EHB complementary to T_23_ on the top strand plays a role in this bias, using a ΔT_23_ mutant site. We found three L-box recombinants out of 642 colonies tested (0.47%) suggesting a role (although minor) for the complementary A base in avoiding L-box recombination of ts *attC* sites. The crossover point in these recombinants could not be determined precisely due to an adenine stretch, but it was not coherent with the cleavage site determined previously for the *attI1* L box. This is probably related to conformational differences between *attI* and *attC* sites that force the crossover to shift. This need for a different crossover point could be itself at the basis of the low frequency of L-box events in the *attI* × *attC* reaction.

## Discussion

In this work we show that integron integrases can perform the second strand exchange in reactions involving exclusively double-stranded sites. We found that integrases recombine the top strand of *attI* sites at frequencies matching those of the bottom strand and that there is no order in the use of strands during the reaction. The crossover point in the top strand of the *attI* site has been elucidated using a novel technique that involves deep sequencing. This approach has allowed overcoming technical difficulties found with classical *in vitro* methods due to the low recombination activity observed. With deep sequencing becoming more accessible, this technique could be useful for others working with recombinases that show low activity or for which optimal conditions *in vitro* have not been elucidated.

Our work reveals surprising aspects of the dynamics of *attI* × *attI* reactions, with broad evolutionary implications that are discussed below. Still, other aspects of the *attI*–IntI interaction remain unclear. *attI* sites are recognized for their sequence, as proven by cognate IntI–*attI* recognition^29^. We (and others previously^38^) have observed in our *in vitro* experiments a cooperative binding starting at the R box. It is also known that secondary recombination sites contain the 5′- AAC -3′ signature of the R box of all *attI* sites^37^. Given that the rest of the R box of the site changes its sequence at every cassette integration event, one could presume that this triplet is fundamental for IntI binding. The almost complete loss of binding activity in our experiments when a mismatch is located in this triplet (Supplementary Fig. 4) supports this idea. Nevertheless, this triplet is universal to all *attI* sites and would not allow integrase discrimination of cognate sites. Also, the sequence of the *attI1* bears a 5′- AAC -3′ triplet at the border of the L box, but the crossover point occurs between different bases (A/A) compared with that of the bottom strand (A/C), suggesting that topology is more important than sequence in the cleavage of the top strand. Therefore, the relative influence of the universal crossover triplet and of the rest of the R box in the binding remains a subject of interest that would need further studying.

This work shows that the avoidance of the second cleavage on *attC* sites is not due to the mere presence of the integrase-specific I2 α-helix, but rather to its interaction with *attC* sites. The extremely low frequencies at which *attC*_*aadA7*_ recombines through the L box highlights the strong constraints that *attC* sites impose on the reaction. It is noteworthy that in natural conditions L-box reactions between *attC* and *attI* sites would place the *attI* site downstream of the cassette. Hence, such tight control on directionality likely serves to ensure the correct functioning of the integron by keeping the *attI* site next to the Pc promoter after consecutive cassette insertions.

From a functional perspective, the reaction between *attI* sites has an unclear adaptive meaning. It could be argued that in chromosomal integrons these reactions have deleterious effects on bacterial fitness, since they lead to the formation of chromosomal dimers that have to be further resolved before cell division. This is probably the reason for the 3–4 orders of magnitude lower recombination frequency for *attI* × *attI* reactions compared with those involving *attC* sites. Nevertheless, in the multicopy world of mobile integrons, one cell can contain more than one integron on the same or different replicons^41^,^42^,^43^. Given the possibility of having Pc's of different strengths, an *attI* × *attI* reaction can lead to the *en bloc* transfer of cassette arrays between integrons. This would alter the expression levels of multiple antimicrobial resistance genes at once, and could have important effects on chemotherapy outcomes. It could be argued that the importance of such rearrangements is probably minor, due to the low recombination frequencies for *attI* × *attI* in laboratory conditions. Nevertheless, one aspect of this reaction that has been constantly overlooked is that, while *attC* sites are normally in their non-recombinogenic form, *attI* sites are recombinogenic during almost the entire cell cycle, with the brief exception of the passage of the replication fork. Despite this low recombination frequency, it is plausible that *attI* × *attI* reactions are more prevalent among mobile integrons than previously envisaged, especially under transient, low-level (or even stochastic) expression of the integrase. These expression pulses typically occur during horizontal gene transfer events^36^,^44^.

This work shows that despite millions of years of evolution towards a system specialized in structured ssDNA recombination, and the acquisition of a 20-residue-long domain within the catalytic core, integron integrases have conserved the activity of their canonical Y-recombinase ancestors. Many examples of DNA breakage and rejoining enzymes acting on dsDNA (like Y-recombinases) or ssDNA (like HUH endonucleases^45^) can be found, but this is the first report of a recombinase with full dual activity on both types of sites, as well as a substrate-dependent switch in recombination pathways. Integrases are capable of recognizing distinct substrates (ds versus ss hairpins) in different ways (sequence versus structure specific) and processing the reaction through different pathways (double strand exchange versus replicative resolution). This duality has deep biological implications, since one pathway is semiconservative and dependent on host machinery, while the other is not. Integrons could function, at least theoretically, using only the classical recombination pathway on dsDNA to deliver cassette integration and excision reactions. It is therefore tempting to speculate that the force driving the evolution of integrons towards a mixed ss/ds-DNA system derives from the benefits of semiconservative recombination. By producing recombined and non-recombined offspring, it allows testing the adaptive value of incoming DNA, while minimizing the deleterious effect of capturing maladaptive genes. Furthermore, it also represents a mechanism for gene duplication, as observed in the *Vibrio cholerae* superintegron^10^ and possibly in some mobile integrons^46^.

From an evolutionary perspective, the functional innovation of integrases towards the recombination of folded ss-*attC* sites, posed important mechanistic constraints in the recognition of the site and the resolution of aHJ to deliver productive reactions. The solution in integrases comes in the form of the additional I2 domain that recognizes the EHBs in *attC* sites. This impedes the second cleavage, allowing for the replicative resolution of the HJ. Interestingly, EHBs and the integrase I2 domain are a good example of coevolution. These structures were probably the first step in the evolutionary transition from the ancestral recombination system to the ss/ds-DNA recombination platform integrons are today. Nevertheless, the acquisition of the I2 domain raises a chicken-egg paradox: how could the domain be selected for in the absence of *attC* sites to provide a gain of function? And how could *attC* sites be selected for before the recognition domain existed? Such a conundrum is found in many evolutionary processes and can easily be understood with the discovery of functional intermediary forms that allow to infer smooth evolutionary transitions between states. We believe integron integrases are representatives of such a bi-functional state. In the light of our results, it now seems possible that an I2-like domain was acquired at a given time by the ancestor of today's integron integrases and, to some degree, permitted the recognition of new ss-substrates while retaining the original activity (or part of it) on the initial site. From this starting point, the streamlining through natural selection, to reach what integrons are today, seems a more straightforward process.

To date, the only recombination reaction that shares some similarities with cassette recombination is the integration of phage CTXφ of *Vibrio cholerae*. Nevertheless, CTXφ cannot be considered a complete system since it does not encode an integrase but instead hijacks the host XerCD recombinases^47^. As a consequence, no specialization of the system towards the recombination of ss-DNA is observed: XerCD lack structural adaptations for the recognition and processing of ss-substrates (I2-like domains), and the phage site stem does not have EHBs but rather mimics a ds-DNA molecule. Nevertheless, the resemblances between cassettes and CTXφ integration, the close phylogenetic relation between integrases and XerCD recombinases and the fact that both systems can coexist in the same bacterial species, makes it tempting to speculate about a viral origin for integrons. Interestingly, the link to the SOS response found in all integrons is also present in many phages, including CTXφ (ref. 48). To date, no chromosome-dimer resolution recombinase of the XerCD family is known to possess a domain similar to I2 and directionality is ensured through their interaction with other host proteins such as FtsK^49^. Nevertheless, one could imagine that acquisition of an I2-like domain by a Xer-like ancestor could have provided the function of avoiding the second strand exchange during phage integration. This would protect the integrity of the chromosome while favouring correct integration events, and would hence be of adaptive value to the cell and the phage. We can now imagine that the chromosome dimer resolution activity of the hybrid recombinase would still be conserved. Phages, or any other integrative mobile element exploiting Xer (IMEX^49^) could have then acted as a vehicle for new adaptive genes, just as they currently do^50^,^51^, representing an ancestor of integron cassettes.

Previous work on evolutionary aspects of Y-recombinases focused on the properties that allow related phage integrases to target specific sequences in the bacterial genome and the possibility of adapting these proteins to recognize non-cognate sites^52^,^53^,^54^. This work sheds light on the evolutionary potential of the Y-recombinase protein fold towards evolutionary innovation (not adaptation). Our results suggest that Y-recombinase-mediated reactions are a more dynamic process than previously assumed. They underscore the robustness of the protein fold, a characteristic that likely confers a high evolvability to the members of this superfamily^6^. It is arguable that the duality in the recognition and binding of integron integrases to the sites has strongly been conserved because cassettes have to be integrated in the *attI* site. Nevertheless, the classical recombination pathway could have been lost through the specialization of integrases towards the single strand exchange pathway, without affecting any of the known aspects of integron dynamics. Therefore, preservation of this pathway after eons of evolution^11^ can be seen as the consequence of a lack of negative selection^55^, or as reflecting an important biological function. The fact that top and bottom strand cleavage of *attI* sites occur at similar frequencies (Fig. 3b) suggests that top strand cleavage is not a promiscuous activity of the integrase and favours the biological function hypothesis. The flexibility of the integrase might not be completely unveiled yet, and functions dependent on some aspects of this plasticity might remain unknown. The second strand exchange resolution pathway opens new avenues for possible mechanisms of cassette genesis, a subject for which no reliable model is yet available^10^.

## Methods

### Bacterial media and conditions

*E. coli* strains were grown in Luria Bertani broth (LB) at 37 °C. Growth at 42 °C was used to induce the expression and force the loss of the plasmid bearing lambda phage integrase. Antibiotics were used at the following concentrations: carbenicillin (Ap), 100 μg ml^−1^, chloramphenicol (Cm), 25 μg ml^−1^, kanamycin (Km), 25 μg ml^−1^, spectinomycin (Sp), 40 μg ml^−1^, Thymidine (Thy) and DAP were supplemented when necessary to a final concentration of 0.3 mM. Glucose and L-arabinose were added at 10 and 2 mg ml^−1^ final concentration, respectively. Chemicals were obtained from Sigma-Aldrich (France).

### Bacterial strains, plasmids and primers

Bacterial strains in this study are *E. coli* DH5α, MG1655 (laboratory collection), П1, and β2163 (ref. 56). Details on strains, plasmids and primers are described, respectively, in Supplementary Tables 1–3.

### DNA procedures

Standard techniques were used for DNA manipulation and cloning^57^. Restriction and DNA-modifying enzymes were purchased from New England Biolabs and Fermentas (Thermo Scientific). PCRs were performed with Dreamtaq DNA polymerase, and Phusion polymerase (Thermo Scientific) according to the manufacturer's instructions. Agarose (1%) electrophoresis gels were used to visualize DNA. DNA purification from PCR products and gels, as well as plasmid extractions, were performed using Qiagen kits. When necessary, DNA sequence was verified using an ABI BigDye Terminator v.3.1 sequencing kit and an ABI Prism 3100 Capillary GeneticAnalyzer (Applied Biosystem). GATC and EUROFINS sequencing services were also used.

### *In silico* work

Protein modelling was performed using Phyre2 (sbg.bio.ic.ac.uk/phyre2) and MacPymol on templates c2a3vA (VchIntIA) and c1ma7A (Cre). Sequence analysis was performed using Geneious 7.1.

### Influence of host machinery in the *attI* × *attI* reaction

To test whether RecA, RecG and RuvABC impact the *attI* × *attI* reaction, we performed a suicide conjugation assay based on that of Biskri *et al*.^35^ and previously implemented in Bouvier *et al*.^17^. Briefly, the *attI* site provided by conjugation is carried on a suicide vector from the R6K-based pSW family that is known to use the Pir protein to initiate its own replication. This plasmid also contains an RP4 origin of transfer (*oriT*RP4). The donor strain β2163 carries the transfer functions in its chromosome, requires DAP to grow in rich medium and can sustain pSW replication through the expression of a chromosomally integrated *pir* gene. The MG1655 recipient strains, which contain the pBAD::*intI1* [Ap^R^] (expressing the IntI1 integrase) and the pSU38Δ::*attI1* [Sp^R^] (carrying the *attI1* site), lacks the *pir* gene and therefore cannot sustain replication of the incoming *attI*-containing pSW vector. The only way for this vector to be maintained in the recipient cell is to form a cointegrate by *attI* × *attI* recombination. We used the ω10001 strain as the donor and as recipient strains the MG1655 *E. coli* ωA197 (control), and its derivatives ω9987 (*recA*-), ω9988 (*recA*-, *recG*-), ω9989 (*recA*-, *ruvABC-)* and ω9990 (*recA*-, *recG*-, *ruvABC*-) (Supplementary Tables 1 and 2). The recombination frequency is calculated as the ratio of transconjugants expressing the pSW marker [Cm^R^] to the total number of recipient clones [Ap^R^, Sp^R^]. Recombination frequencies correspond to the average of at least three independent trials. The *attI* × *attI* co-integrate formation was checked by PCR with the appropriate SWbeg and MFD primers (on eight randomly chosen clones per experiment).

### Mismatched covalent circles preparation

Construction of the p8669 and p8670 phagemid vectors to produce mismatched covalent circles was performed as in ref. 21 with some modifications. Briefly, we exchanged *attC* sites in plasmids p7770 and p7771 for *attI1* sites, giving rise to plasmids p8669 and p8670 (plasmids, carrying the *oriFD* in both orientations ensuring the production of respectively the bottom (*oriFd1*) and top strand (*oriFd2*)). Single strand DNA production was performed using M13K07 Helper Phage as in ref. 21. Both phagemid vectors (p8669 and p8670) are introduced by transformation into *F′* carrying *pir* strain cells (β2150, ω4446) to respectively obtain the ω8675 and ω8676 strains. M13 infection and purification of ssDNA were performed according to the manufacturer (M13K07 Helper Phage and QIAprep Spin M13 kit). The complementary single strand DNA molecules were annealed, digested by both EcoRI and MfeI restriction enzymes to eliminate *oriFd* (which does not anneal due to the inverted orientation in each molecule), and self-ligated.

### Non-replicative recombination assay

This assay was developed previously in the laboratory^58^. We transform with 1–2 μg mismatched covalent circles containing the *attI* site into MG1655*mutS215* strain containing the pBAD::*intI1* (p3938) and the pSU38Δ::*attI1* (p929) plasmids (strain ω7994). Circles cannot replicate in this genetic background (ω7994). Note that this assay was first envisaged in a *mutS*-, *recA*-strain but transformation efficiency was too low due to a filamenting (sick) phenotype in the double mutant. We hence studied the influence of *recA* and found it to be responsible for <0.5% of all recombinants in our assay (see below recombination frequencies with and without pBAD::*intI1*). This justified the use of a *recA*+ strain neglecting the influence of homologous recombination in the recombination process. Competent cells were prepared in the presence of 0.2% arabinose to allow integrase expression. Selection on chloramphenicol (Cm)-containing plates (the mismatched circles marker) yielded, almost exclusively, *attI* × *attI* recombination events. The *attI* × *attI* cointegrate formation was checked on 101 clones by PCR with SeqattI and SeqNar primers. These PCR products include the mismatch-containing region, and were analysed for their resistance or sensitivity to both SacII and NarI restriction enzymes.

As a control, we performed a similar experiment using an MG1655*mutS215* strain containing the pSU38Δ::*attI1* but lacking pBAD::*intI1* (strain ωA266). To establish a recombination frequency, we transformed in parallel the same *mutS* strain lacking both the pSU38Δ::*attI1* and pBAD::*intI1* plasmids but containing a Pir-expressing plasmid (pSB118::*pir116*, p1177) ensuring the replication of the incoming circle (strain ω7120). Recombination activity corresponds to the ratio of Cm^R^ clones obtained in *pir−* conditions (with and without integrase) to those obtained in *pir+* conditions. Note that the efficiency of transformation of each strain was determined beforehand and used to adjust the final ratio and normalize the results. We obtained a recombination frequency of 1.17 × 10^−3^ in presence of integrase, and of 4.24 × 10^−6^ in the absence of integrase.

As a supplementary control, we analysed the Cm^R^ clones obtained in *pir+* context (Supplementary Fig. 2). After pooling of 319 clones, plasmids were extracted and retrotransformed in П1 *pir+* competent cells (ω1628). Then, 101 transformants were analysed by SacII and NarI restriction and/or by sequencing (using SeqNarI and SeqSacII primers). The segregation pattern was, as indicated in Supplementary Fig. 2, of 49.5% NarI+/SacII−, 48.5% NarI−/SacII+, 1% of NarI+/SacII+ and 1% NarI−/SacII−. This close-to-50% segregation confirms the correct stoichiometry of each strand, and validates the production of the mismatched circles.

### Chromosomal inversion test proves a second strand exchange

**Figure 2:**
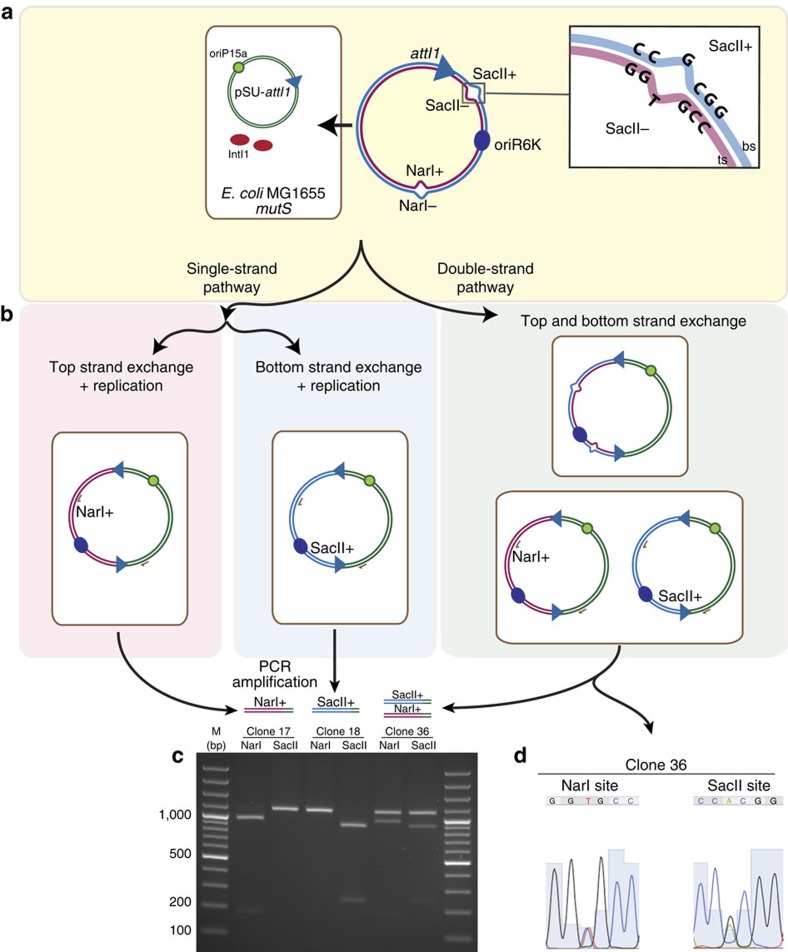
The top strand of *attI1* is reactive. (**a**) Experimental set-up used for mismatched-covalent-circles recombination. Mismatched circles bearing an *attI1* site are transformed in an *E. coli* MG1655 *mutS*^*−*^ strain bearing a pSU-*attI1* plasmid and expressing the integrase in trans. (**b**) Representation of the recombination products resulting from the three possible HJ resolution pathways. The mismatch-bearing regions were PCR amplified and subjected to NarI and SacII restriction. (**c**) Restriction pattern analysis of three clones obtained, representing the three resolution pathways. (**d**) Sequencing of clone 36 PCR product revealed the expected mismatches.

**Figure 3:**
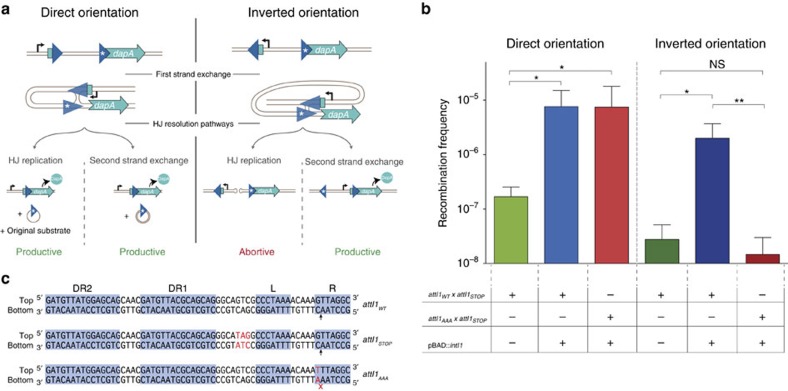
The integrase can resolve HJs through second strand exchange. (**a**) Graphic representation of the structural set-up of our experiment in which an *attI1* × *attI1* reaction reconstitutes the *dapA* gene, with *attI1* sites in a direct orientation (left column) and in an inverted orientation (right column). Asterisks indicate *attI* sites modified to include a STOP codon (*attI1*_STOP_). The topology of HJs resulting from the first strand exchange is shown for both orientations. Recombination products following the two possible HJ resolution pathways are shown below. (**b**) Recombination frequency of all set-ups. Columns represent the mean and error bars the s.d. (*n*⩾3). Significance testing was performed with Kolmogorov–Smirnov test (*P value <0.05; **P value <0.01; NS, not significant). For the direct orientation set-up, strains used are B36, B82 and C162 (first, second and third column, respectively). For the inverted orientation set-up, strains used are B37, B83 and C163 (fourth, fifth and sixth column, respectively); their relevant genotypes are described in the table beneath the graph. (**c**) Sequence of the *attI* sites used in this experiment: *attI1*_WT_ (the cleavage point on the bottom strand is marked with an arrow), *attI*_STOP_, a variant not encoding an ORF, that allows to reduce background noise in the experiment while remaining recombinogenic; and *attI*_*AAA*_, a variant in which the bottom strand is inactive.

In this experiment we used an assay in which recombination between two sites leads to the reconstitution of the essential *dapA* gene allowing for growth in media not supplemented with DAP^28^,^36^. DNA molecules containing the sites in direct orientation were designed *in silico* and synthesized by GeneART (Life Technologies). To avoid background noise (expression and translation of *dapA*) before recombination, the right *attI* site (the one immediately upstream *dapA*) was modified to include a STOP codon 1 bp upstream of the L box (Fig. 2). The design also included two XhoI and SmaI restriction sites surrounding the left *attI* site and the P_ndmA_ promoter (registry part J23100) that drives transcription of *dapA* after recombination. Through XhoI/SmaI double digestion we inverted the left site to obtain the inverted orientation. These constructions were subsequently cloned EcoRI/NruI into the pir-dependent plasmid pA669 (Sp^R^) containing a lambda phage *attP* site, and transformed in П1 *pir+* competent cells (ω1628). A FRT recombination site was added in EcoRI to allow for the resolution of dimers after integration in the chromosome. Sequence was verified and these plasmids were transformed in the ω8488 strain, an MG1655 *recA*- *dapA*- *E. coli* strain containing the replication/expression-thermosensitive plasmid p3153 bearing the lambda phage integrase (Ap^R^). In this strain, *pir*-dependent plasmids do not replicate and are only maintained through their integration in the lambda *attB* site of the chromosome. Integration events where selected for using the Sp resistance marker of the plasmid. Loss of the p3153 plasmid after overnight incubation at 42 °C was verified by replica plating on media containing carbenicillin (selecting for the Ap^R^ marker of p3153). Monomeric integration was verified through PCR with primers (1329 and 1698). Sequence was verified in all strains produced. Strains ωB36 and ωB37 containing the constructions in direct and inverse orientation were selected for the study and transformed with the pBAD::*intI1* (p3938) plasmid giving rise to strains ωB82 and ωB83. To test the recombination rate of the genetic structures, strains were grown overnight in LB supplemented with DAP, carbenicillin, spectinomycin and glucose, then diluted 1:50 into fresh media containing DAP, carbenicillin (to avoid the loss of p3938) and glucose or arabinose to repress or induce the expression of the *intI1*, and incubated for 3 h at 37 °C with shaking. Cultures were then plated on selective media with and without DAP. The recombination frequency is calculated as the ratio of colonies growing without DAP (recombined) to the total number of colonies growing on DAP-supplemented media. Recombination frequencies correspond to the average of at least three independent assays.

### Killing the bottom strand

To obtain constructions in which the left recombination *attI* site bore a C to A mutation in the cleavage site, we performed site-directed mutagenesis through overlap extension PCR combining primers attI CxA R and attI GxT F with primers XhoI F and XhoI R and using pA873 as template. The PCR product was cloned in both orientations in plasmid pA873 using XhoI restriction sites, giving rise to plasmids pC131 and pC132. These plasmids were inserted in the chromosome of ω8488 as described above and verified clones (strains ωC139 and ωC140) were further transformed with the pBAD::*intI1* (p3938) plasmid to obtain strains ωC162 and ωC163. Recombination rates were measured as above.

### Determining the crossover point

**Figure 4:**
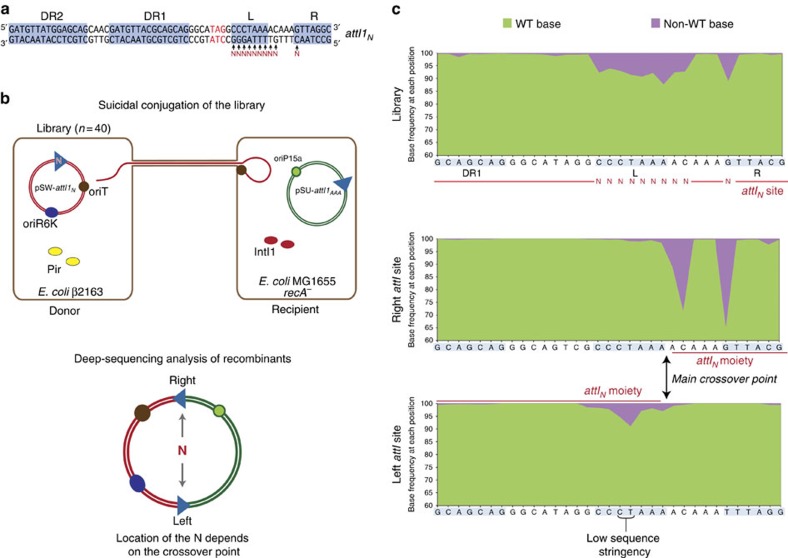
Mapping of the crossover point on the top strand. (**a**) Positions randomized in the *attI*_STOP_ site to produce the *attI*_*N*_ library (*n*=40). The theoretical frequency of bases in every randomized position is of 92.5% WT base and 2.5% of every other base. (**b**) Diagram of the experimental set-up and recombination reaction. Recombination between *attI*_*N*_ and *attI*_*AAA*_ takes place through the top strand, splitting the randomized bases between moieties in the resulting sites of the cointegrate (right and left). (**c**) Graphical representation of base frequency within the *attI*_*N*_ library before recombination, and the right and left sites after recombination. Green, WT base; Purple, non-WT base.

**Figure 5:**
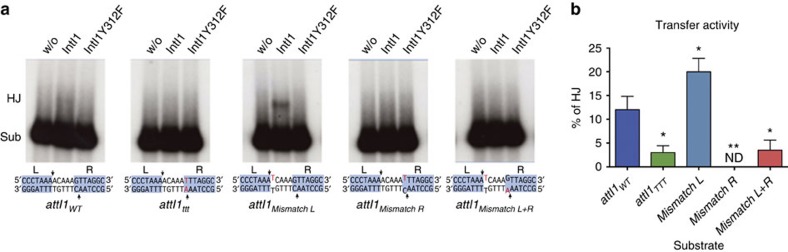
Mismatches in the top strand abolish HJ resolution *in vitro*. *In vitro* activity of IntI1 on different *attI* substrates. (**a**) Gel Strand transfer test allowing the observation of HJ intermediates. Resolution of these structures through a second cleavage converts them back to the original substrate (sub). A diagram of the L and R boxes of the site is shown below its cognate gel (the rest of the site is identical to the wild type in all cases), where red bases represent changes with respect to the wild-type sequence. Crossover points in both strands are indicated with black arrows. (**b**) Transfer activity quantified as the amount of signal retained in the filter (HJs) over the total amount of signal. Columns represent the mean and error bars the s.d. (*n*=3). Transfer activity for IntI1_Y312F_ was undetectable in all assays. Statistical analysis was performed using Dunnett's test with the activity on the WT site as the control (*significant (*α*=0.05); ^**^, significant (*α*=0.01); ND, not detected).

To build the library of *attI*_*N*_ sites in the *pir*-dependent pSW plasmid we combined primer 2915 with primers 2916–2925, obtaining 10 *attI*_*N*_ sites, each with a random base at a given position. These sites were mixed, digested XhoI, PstI, cloned into the pSW plasmid pC066 (Sp^R^) and transformed into П1 *pir+* competent cells (ω1628). >5,000 transformants were mixed to establish the library (ωC351). Sequencing of random clones showed that 18 out of 20 had bases other than WT in the expected positions, validating the library (see also the deep sequencing results in Fig. 4). The library was subcloned into a β2163 donor strain to be deliverable through conjugation (ωC373).

To force L-box recombination, the *attI* site on the resident plasmid was changed to an *attI*_*AAA*_ site using primers 2889 and 2932 and cloned EcoRI/BamHI in p929 (Km^R^), giving rise to pC252. Strain ω4826 (MG1655 *recA*^−^) was transformed with pC252 and p3938 (pBAD::*intI1*) (ωC307). As controls of this experiment we included a strain transformed with p929 instead of pC252 (ωC305) and strains without the p3938 (ωC304 (ω4826/p929) and ωC306 (ω4826/pC252)).

The library was delivered through conjugation from ωC373 to ωC307. Recombination frequency was of 2.63 × 10^−4^ in the presence of arabinose (inducing the expression of the integrase), and of 1.88 × 10^−6^ in the presence of glucose, confirming the IntI1-mediated recombination of pSW-*attI*_*N*_ into pSU-*attI*_*AAA*_. More than 7,000 colonies from the conjugation assay were collected (ωC453 to ωC455) and subjected to plasmid extraction. As a control, conjugation assays with ωC373 and ωC305 (pSU-*attI*) were performed, showing similar recombination frequencies: 1.64 × 10^−4^ with arabinose and 2.59 × 10^−6^ with glucose.

The *attI* sites from the library and the recombination products were PCR amplified using primers 1236-attIN's right (library); and 1236-738 and MRV- attIN's right (recombinant sites). PCR products were used for sequencing on an Illumina MySeq. Resulting sequences were filtered to discard abnormal recombination events and further analyzed using Galaxy (https://usegalaxy.org) and IGV 2.3 (https://www.broadinstitute.org/igv/home). Base frequency was analysed at every position in the library and the recombinant sites. The expected frequency of bases in each randomized position is of 92.5% of wild-type base and 2.5% of every other base. This is because for a given position only 1 out 10 sites bears a random base (90% WT-10% N) and 25% of random bases are wild type (92.5% WT and 7.5% not WT). The observed base frequency composition in the library before recombination matched the expected distribution. Sequencing of the resulting sites after recombination showed a split distribution of randomized bases in both sites, allowing to infer the crossover point.

### *In vitro* experiments

Translational fusions with a maltose-binding protein were obtained of IntI1 and the catalytically inactive IntI1_Y312F_ by cloning in the commercial vector pMAL-CX5 and transforming *E. coli* Top10 strain. Plasmids were further purified and transformed in the BL21 strain for protein production. Colonies from this transformation were collected and grown overnight in 25 ml of LB containing carbenicillin and 0.2% glucose. After incubation, cells were collected by centrifugation at 4 °C, resuspended in 25 ml of fresh media, and inoculated in 3 l of LB containing carbenicillin. When the culture reached an OD= 0.6, IPTG was added at 0.3 mM and the culture was incubated at 14 °C overnight. The cells were then collected by centrifugation and frozen. Cells were thawed to room temperature under cold tap water, and lysed by passage through an Emulsiflex C-5 cell breaker (Avestin Europe GmBH) at 13,000 p.s.i. at 4 °C. The cell lysate was centrifuged at 23,500*g* for 40 min in an RC-5C using an ss-34 rotor (Sorvall, Thermo Scientific). The IntI1-MBP fusion protein was purified with 5 ml Amylose column (GE Healthcare) on an AKTA-prime FPLC machine (GE Healthcare).

The sequence of the oligonucleotides used for the strand transfer assay is reported in Supplementary Table 3. A total of 100 pmol of oligonucleotides coding for top and bottom strands were labeled using T4 DNA kinase (Promega) and 25 mCi ^32^P gamma ATP for 1 h at 37 °C. Once radiolabelled, top and bottom strands were then annealed (85 °C for 3 min and at room temperature for overnight). The strand transfer assay was performed using conditions derived from refs 40, 59. Briefly, purified MBP-IntI1 was diluted in 1 M NaCl, 20 mM HEPES, 1 M, pH 7, 10 mM DTT for 30 min on ice. Then incubated with the different recombination sites (25 nM final) for 30 min on ice in a final volume of 5 μl. The reaction was then started by adding 5 μl of the reaction buffer (final concentrations 100 nM IN, 15% DMSO, 8% PEG, 10 mM MgCl_2_, 100 mM NaCl, 10 mM DTT). Samples were incubated for 2 h at 37 °C. After reaction the products were treated 1 h at 55 °C with proteinase K (Promega) 1 mg ml^−1^ final concentration and deproteinized using phenol/choloroform/isoamyl-alcohol (24/25/1 v/v/v). Aqueous phase was run on 12% polyacrylamide gel at 1,000 V for 5–6 h. Gel was then autoradiographied and quantified using Image J software (Supplementary Fig. 4b).

A total of 5 pmol of MBP-IntI1 were incubated for 20 min at room temperature with 1 pmol of radiolabelled double-stranded DNA containing the recombination site sequence in 15% DMSO, 8% PEG, 10 mM MgCl_2_, 100 mM NaCl, 10 mM DTT. The complexes were then filtered on nitrocellulose filters. Filters were washed three times with PBS and counted using a Wallac 1409 liquid scintillation counter.

### L-box recombination in the *attC*_*aadA7*_ × *attI1* reaction

This experiment is based on the suicidal conjugation assay explained above. This time the plasmids delivered by conjugation carry the top or bottom strand of the wild type or the ΔT23 *attC*_*aadA7*_ sites. Maintenance of these plasmids in the recipient cell is only possible through an *attC* × *attI* reaction. We used the ωD060, ωD061, ωD805 and ωD806 strains as donors and ω9669 as the recipient strain. The recombination frequencies in R′ (bs) and R′′ (ts) and the corresponding mean deviations were deduced by determining the orientation of cassette integration for at least 20 independent clones for each bs transfer (and multiplying the total recombination frequency by the ratio of R′ and R′′ recombination) and for 100 clones in the ts transfer assays. The orientation of cassette integration was determined by performing two PCR reactions: the first reaction with SW23begin/MFD primers produced a product only when the recombination took place in the delivered *attC* strand (in R′ when bs is delivered, 530 bp; in R'' when ts is delivered, 520 bp); the second reaction with SW23end/MFD primers produced a product only when the recombination took place in the resynthesized *attC* strand (in R′′ when bs is delivered, 240 bp; in R′ when ts is delivered, 210 bp). In addition, the recombination in the L box was detected by performing two PCR reactions on all clones. The first reaction with RpLp/571 produced a product when the recombination took place in the L′ box (340 bp); the second reaction with RppLpp/571 produced a product when the recombination took place in the L′′ box (340 bp). Recombination through the L′L′′ box of *attC* sites was only detected when the top strand was delivered (data for bs is hence not shown).

## Additional information

**Accession codes**: The crossover point sequencing data for the library, left site and right site has been deposited at the Sequence Read Archive under the accession code SRP071193.

**How to cite this article:** Escudero, J. A. *et al*. Unmasking the ancestral activity of integron integrases reveals a smooth evolutionary transition during functional innovation. *Nat. Commun.* 7:10937 doi: 10.1038/ncomms10937 (2016).

## Supplementary Material

Supplementary Information Supplementary Figures 1-5, Supplementary Tables 1-3 and Supplementary References

Supplementary Movie 1 Replicative resolution of the HJ formed by attI1 sites in inverted orientation. In this orientation, the replicative resolution of the HJ after only one strand exchange linearizes the replicon and is abortive.

Supplementary Movie 2 Classical resolution pathway of the HJ formed by attI1 sites in inverted orientation. Recombination between attI1 sites in inverted orientation is productive if resolved through a second strand exchange. The DNA segment between both sites is inverted after recombination, as highlighted by the color code of both strands.
