# Supplementary material for: Unmasking the ancestral activity of integron integrases reveals a smooth evolutionary transition during functional innovation
Source: Nat Commun. 2016 Mar 10;7:10937. doi: 10.1038/ncomms10937 (PMC4792948; doi:10.1038/ncomms10937)
Supplement: Supplementary Information — Supplementary Figures 1-5, Supplementary Tables 1-3 and Supplementary References [file ncomms10937-s1.pdf]

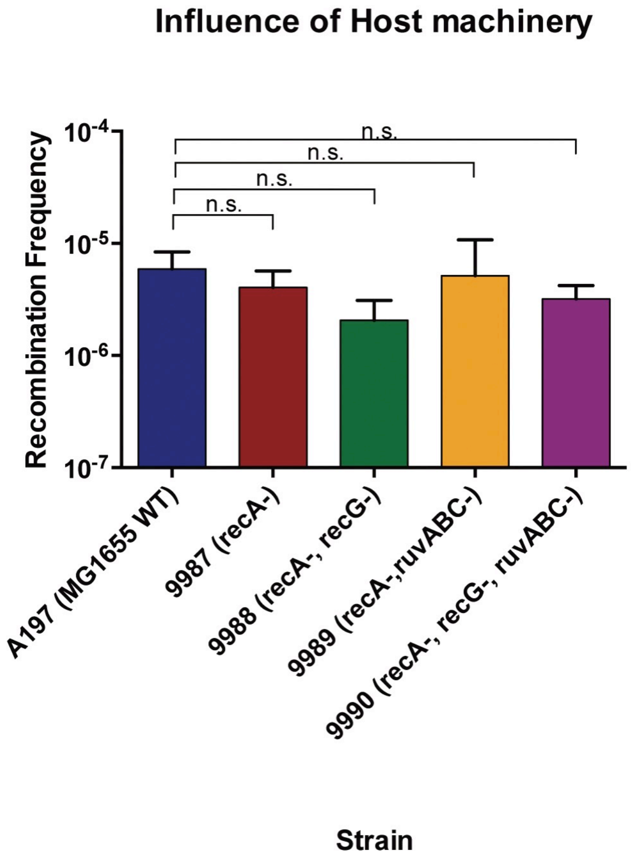

Supplementary Figure 1. Influence of host machinery involved in HJ resolution, in the *attI* x *attI* reaction. Bar graph representing the recombination frequency of an MG1655 parental strain and *recA*-, *recG*-, *ruvABC*- and the triple mutant derivatives thereof. Columns represent the mean and error bars represent standard deviation (n=3). Significance testing was performed with Kolmogorov-Smirnov test (P value < 0.05; n.s. not significant.)

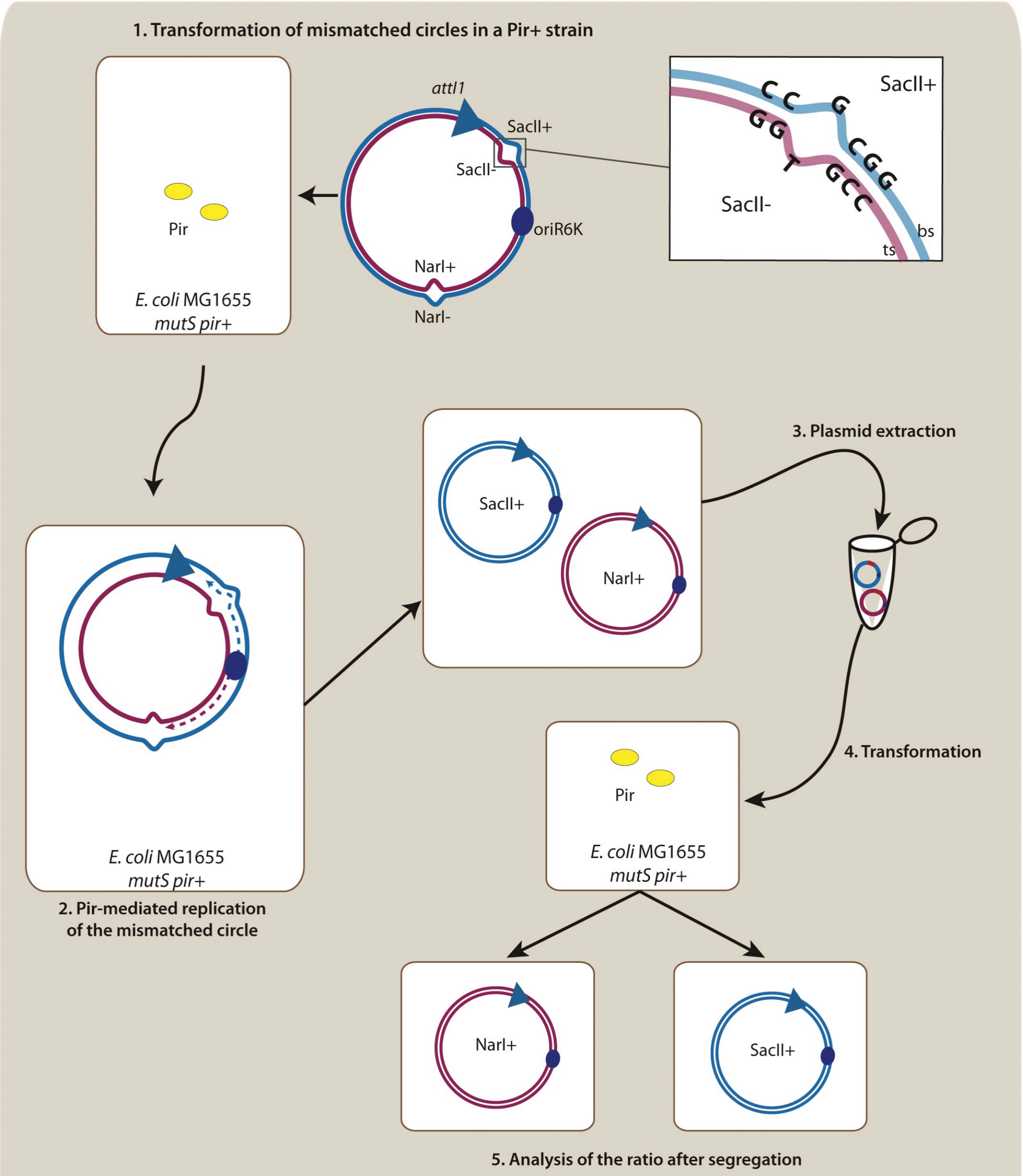

	SacII$^-$/NarI$^+$	SacII$^+$/NarI$^-$	SacII$^+$/NarI$^+$	SacII$^-$/NarI$^-$
**Number of clones** Total =101	50	49	1	1
**Percentage**	49,5%	48,5%	1%	1%

Supplementary Figure 2. Verification of mismatched covalent circles. **1**, After production and hybridization of the two strands (see "materials and methods"), the mismatched covalent circles were transformed into an MG1655 *mutS*$^-$, *pir*$^+$ strain. In this genetic background circles are theoretically not repaired (*mutS*$^-$) and can be replicated (*pir*$^+$). **2**, Each strand is hence used as a template for replication, leading to a mixed population of plasmids within the cell. If the construction of the circles is correct and the 1:1 stoichiometry between strands is respected, each profile should represent 50% of the plasmid content within the cell. **3**, To analyze this, plasmids were extracted, diluted and segregated again by transformation in the same strain (**4**). **5**, Transformants were analyzed by PCR amplification and restriction, giving a close to 50/50 distribution of profiles.

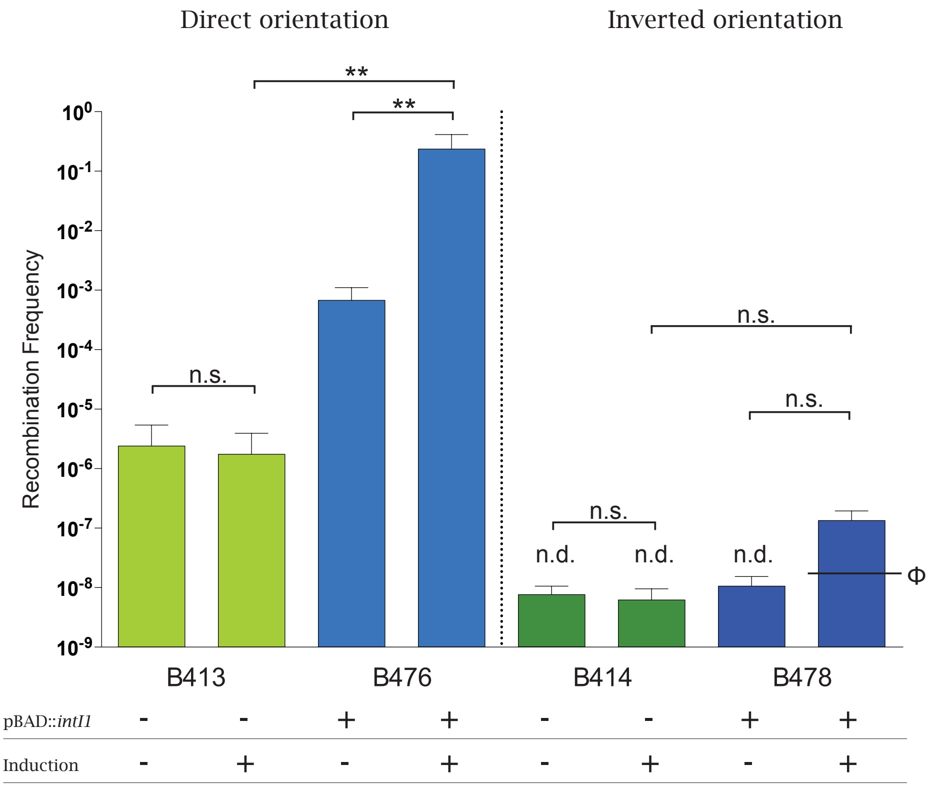

Supplementary Figure 3. Recombination frequency of *aadA7* x *ereA2 attC* sites in direct and inverted orientation. Columns represent the mean and error bars represent standard deviation (n≥3). Significance testing was performed with an ANOVA test (**: P value < 0.01; n.s: not significant; n.d.: not detected). In the reactions where no recombinants were detected the column represents the limit of detection of the experiment. Φ represents the limit of detection ($1,67x10^{-8}$) for recombination events in which the *attC* could have hypothetically been recognized and processed as a double strand (not detected).

a

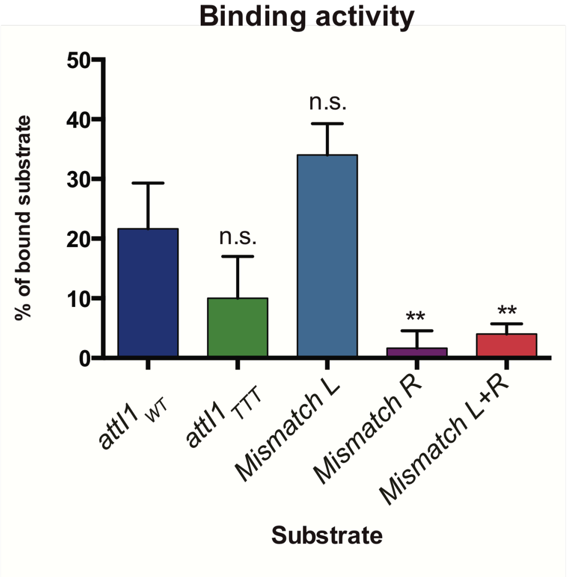

b

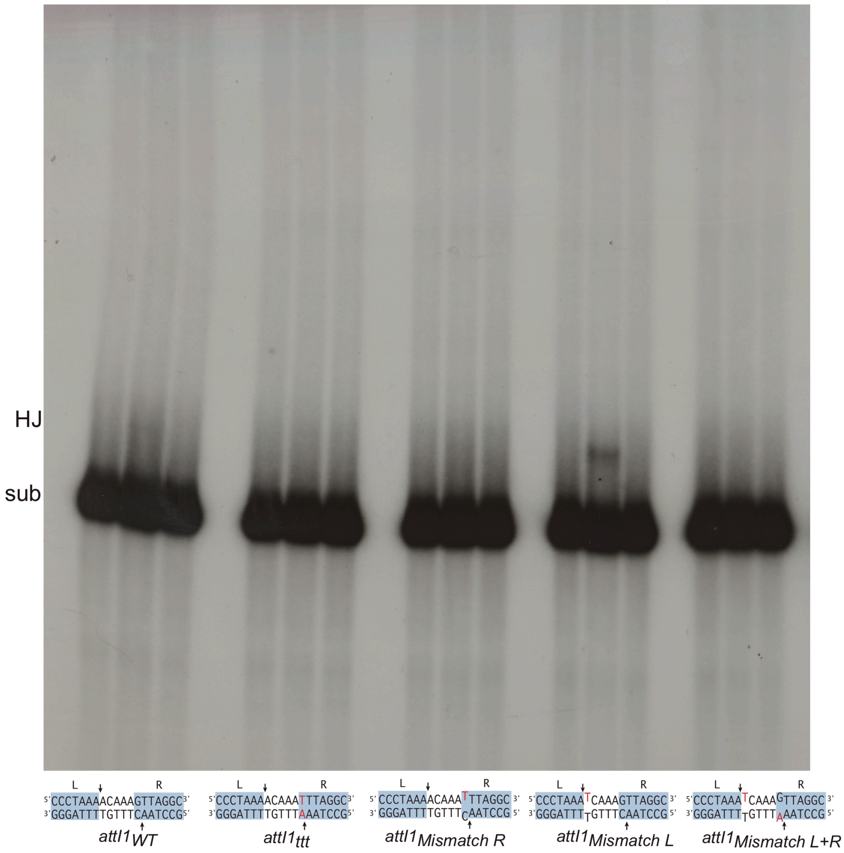

Supplementary Figure 4. Activity of IntI1 on mismatched sites **(a)** Binding activity of IntI1 to wt *attI1* site and mismatched sites. Binding is measured as the percentage of substrate retained on nitrocellulose filters after incubating 5 pmoles of integrase with 1 pmol of radiolabeled substrate. Values represent the mean and error bars the standard deviation (n=3). Statistical analysis was performed using Dunnett's test with the binding to the wt site as the control (n.s.: not significant; **: significant (α=0,01)). **(b)** Uncut gel from Figure 5. Sub: substrate.

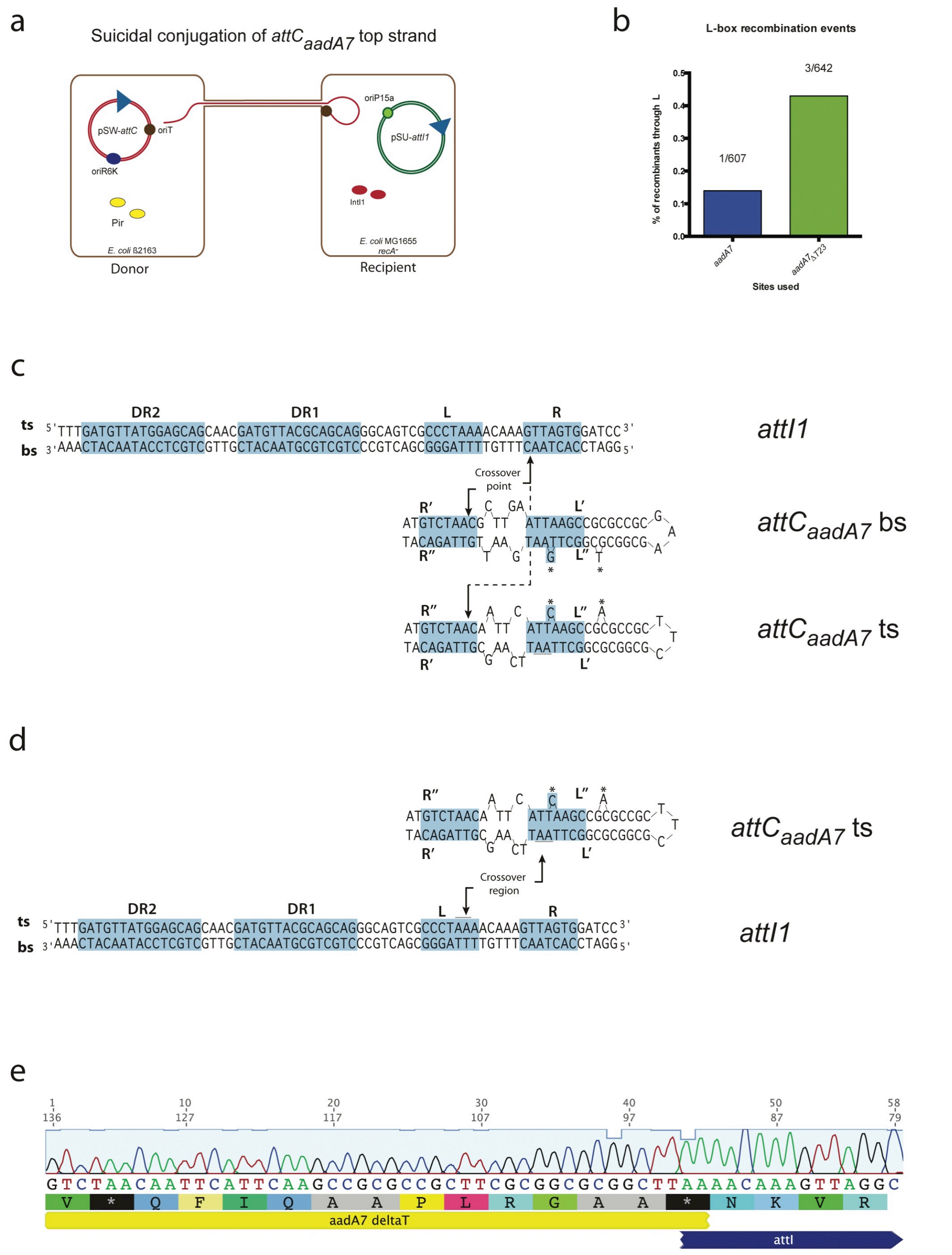

Supplementary Figure 5. L-box recombination in the *attI* x *attC* reaction. (**a**) Diagram of the suicidal conjugative delivery of the top strand of *attC*$_{aadA7}$ and a ΔT_{23} variant. (**b**) Frequency of L-box recombination events (described in **d**) for wild type and ΔT_{23} mutant site. (**c**) Diagram of the known recombination events involving the R box of *attC*$_{aadA7}$ (bs and ts) and of *attI*. (**d**) Recombination events through the L-boxes detected in this experiment. The crossover region is marked based on **e**. (**e**) Example of the sequence of the *attI1* x *attC*$_{aadA7\Delta T23}$ recombination product after left-box recombination. The exact crossover point cannot be determined due to adenine repetitions.

Supplementary Table 1. *Escherichia coli* strains used in this work

Strain number	Relevant genotype	Reference
**Basic Strains**		
ω55	*E. coli* K12 MG1655	Laboratory collection
ω162	DH5α	Laboratory collection
ω1628	Π1 DH5α *ΔthyA*::(erm-pir)	Demarre *et al.* 2005[1]
ω72	β2163	Demarre *et al.* 2005[1]
ω87	β2150 Δ*dapA*::(*erm,pir*) *thrB1004, pro, thi, strA, hsdS, lacZ* D*M15*,(F' *lacZ* D*M15 lacIq, traD36, proA+, proB+*)	Demarre *et al.* 2005[1]
ω8488	MG1655 Δ*dapA* recA269::Tn10 (p3153) strain used for the integration of pSW plasmids into the attB site of the chromosome through lambda recombination mediated by plasmid p3153	This work
**Host Machinery**		
ωA197	MG1655 (p3938) (p4884)	This work
ω9987	MG1655 recA269::Tn10 (N4279) with plasmids p3938 and p4884	RG. Lloyd and this work.
ω9988	MG1655 recA269::Tn10 recG263::kan (N5059) (p3938) (p4884)	RG. Lloyd and this work.
ω9989	MG1655 recA269::Tn10 ruvABC::cm (N5091) (p3938) (p4884)	RG. Lloyd and this work.
ω9990	MG1655 recA269::Tn10 ruvABC::cm recG263::Km (N5070) (p3938) (p4884)	RG. Lloyd and this work.
**Mismatched Covalent Circles**		
	**Single strand production**	
ω8675	β2150 (p8669)	This work
ω8676	β2150 (p8670)	This work
	**Transformed strains**	
ω7120	MG1655*mutS*215 (p1177)	Loot *et al.* 2012[2]
ω7994	MG1655*mutS*215 (p3938) (p929)	Loot *et al.* 2012[2]
ωA266	MG1655*mutS*215 (p929)	Loot *et al.* 2012[2]
ω1628	Π1 DH5α *ΔthyA*::(erm-pir)	Demarre *et al.* 2005[1]
**Chromosome recombination assay**		
ωA642	DH5α (pA642) plasmid from GeneArt with *attI* sites in direct (head to tail) orientation.	This work
ωA749	DH5α (pA749) inversion of the left *attI* site in pA642 (*attI* sites in tail to tail orientation)	This work
ωA873	Π1 (pA873) cloning of $attI1_{WT}$-$attI1_{STOP}$-*dapA* in direct orientation (from pA642) in a pSW plasmid	This work
ωA874	Π1 (pA874) cloning of $attI1_{WT}$-$attI1_{STOP}$-*dapA* in inverse orientation (from pA749) in a pSW plasmid	This work
ωB36	ω8488 attB::$attI1_{WT}$-$attI1_{STOP}$-*dapA* [Sp^R] (integration of plasmid pA873 in the chromosome). Direct orientation of the sites.	This work
ωB37	ω8488 attB::$attI1_{WT}$-$attI1_{STOP}$-*dapA* [Sp^R] (integration of plasmid	This work

	pA874 in the chromosome). Inverse orientation of the sites.	
ωB82	B36 (p3938)	This work
ωB83	B37 (p3938)	This work
ωC131	Π1 (pC131) plasmid in which the $attI_{WT}$ of pA874 has been changed for an $attI_{AAA}$)	This work
ωC132	Π1 (pC132) plasmid in which the $attI_{WT}$ of pA873 has been changed for an $attI_{AAA}$)	This work
ωC139	ω8488 attB::$attI1_{AAA}$-$attI1_{STOP}$-dapA [Sp^R] (integration of plasmid pC132 in the chromosome). Direct orientation of the sites.	This work
ωC140	ω8488 attB:: $attI_{AAA}$ -$attI1_{STOP}$-*dapA* [Sp^R] (integration of plasmid pC131 in the chromosome). Inverse orientation of the sites.	This work
ωC162	ωC139 (p3938)	This work
ωC163	ωC140 (p3938)	This work
ωB413	ω8488 attB:: $attC_{aadA7}$ –$attC_{ereA2}$-*dapA* [Sp^R] (integration of plasmid pB350 in the chromosome). Direct orientation of the sites.	This work
ωB414	ω8488 attB:: $attC_{aadA7}$ –$attC_{ereA2}$-*dapA* [Sp^R] (integration of plasmid pB340 in the chromosome). Inverse orientation of the sites.	This work
ωB476	ωB413 (p3938)	This work
ωC478	ωB414 (p3938)	This work
	**Cleavage point determination through Deep Sequencing**	
ωC351	Π1 containing the library of $attI_N$ plasmids (pC351)	This work
ωC373	β2163 containing pC351 library.	This work
ωC307	MG1655 *recA* (p3938)(pC252)	This work
	**IntI1-MBP purification**	
ω9958	Top10 strain bearing pMAL-CX5::$intI1_{Y312F}$	This work
ωB335	Top10 strain bearing pMAL-CX5::*intI1*	This work
ω888	*E. coli* BL21 (DE3) strain containing the T7 polymerase and plasmid pLysS expressing T7 lysozyme	Studier *et al.* 1990[3]
	**Left box recombination in the *attC* x *attI* reaction**	
ωD060	β2163 donor of pSW23T::$attC_{aadA7}$ bs	This work
ωD059	β2163 donor of pSW23T::$attC_{aadA7}$ ts	This work
ωD805	β2163 donor of pSW23T:: $\Delta T_{23}attC_{aadA7}$ bs	This work
ωD806	β2163 donor of pSW23T:: $\Delta T_{23}attC_{aadA7}$ ts	This work
ω9669	Recipient DH5α bearing p3938 (pBAD::*intI1)* and p929 (pSU38Δ::*attI1)*	This work

Supplementary Table 2. Plasmids used in this study

Plasmid Number	Relevant properties and construction
	General plasmids
p929	pSU38Δ::*attI1*, ori_{p15A} [Km^R][4]
p3938	pBAD::*intI1*, oriColE1 [Ap^R][5]
p4884	pSU38Δ::*attI1*, ori_{p15A} [Sp^R][2]
pSW23T	plasmid dependent on the pi protein for replication
pMAL-C5X	plasmid for protein purification using a maltose binding protein tag (commercial)

Mismatched covalent circles	
p8669	pSW24:: *attI1*, *oriFd1*, SacII^{+} NarI^{-} $oriV_{R6K}$, [Cm^{R}]. EcoRI/BamHI substitution of the *attC* site of p7770[2] by an attI1 site.
p8670	pSW24:: *attI1*, *oriFd1*, SacII^{-} NarI^{+} $oriV_{R6K}$, [Cm^{R}]. EcoRI/BamHI substitution of the attC site of p7771[2] by an attI1 site.
p1177	pSB118::*pir116*[1]
Double cleavage. Chromosome.	
pA642	pMK-RQ:: :: $attI1_{WT}$-$attI1_{STOP}$-$\Delta dapA$ (dir), *ColE1* (Km^{R}). Plasmid from GeneArt.
pA749	pMK-RQ:: :: $attI1_{WT}$-$attI1_{STOP}$-$\Delta dapA$ (inv), *ColE1* (Km^{R}). Inversion of the left *attI* site in pA642by SmaI/XhoI digestión and religation.
pA873	pSW23T:: $attI1_{WT}$-$attI1_{STOP}$-*dapA* (dir), $oriT_{RP4}$, $oriV_{R6K}$, *attP* [Sp^{R}]. Insertion (EcoRI/NruI) from pA642 into pSW23T. FRT (Flippase Recognition Target) added in EcoRI.
pA874	pSW23T:: $attI1_{WT}$-$attI1_{STOP}$-*dapA* (inv), $oriT_{RP4}$, $oriV_{R6K}$, *attP* [Sp^{R}]. Insertion (EcoRI/NruI) from pA749 into pSW23T. FRT (Flippase Recognition Target) added in EcoRI.
pC131	pSW23T:: $attI1_{AAA}$-$attI1_{STOP}$-*dapA* (dir), $oriT_{RP4}$, $oriV_{R6K}$, *attP* [Sp^{R}]. Modification of the left *attI* site from pA873 to avoid bottom-strand recombination.
pC132	pSW23T:: $attI1_{AAA}$-$attI1_{STOP}$-*dapA* (inv), $oriT_{RP4}$, $oriV_{R6K}$, *attP* [Sp^{R}]. Modification of the left *attI* site from pA874 to avoid bottom-strand recombination.
pB350	pSW23T:: $attC_{aadA7}$-$attC_{ereA2}$-*dapA* (dir), $oriT_{RP4}$, $oriV_{R6K}$, *attP* [Sp^{R}]. FRT (Flippase Recognition Target) added in EcoRI.
pB340	pSW23T:: $attC_{aadA7}$-$attC_{ereA2}$-*dapA* (inv), $oriT_{RP4}$, $oriV_{R6K}$, *attP* [Sp^{R}]. FRT (Flippase Recognition Target) added in EcoRI.
Cleavage site: Deep sequencing	
pC351	pSW23T::$attI1_{N}$ Library of plasmids bearing random bases in *attI1* (XhoI/PstI).
pC252	pSU38 bearing $attI_{TTT}$.
attC x attI recombination through the L box.	
pD060	pSW23T::$attC_{aadA7}$ bs
pD059	pSW23T::$attC_{aadA7}$ ts
pD805	pSW23T:: $\Delta T_{23}attC_{aadA7}$ bs
pD806	pSW23T:: $\Delta T_{23}attC_{aadA7}$ ts

Supplementary Table 3. Oligonucleotides used in this study

Number	Name	Sequence	Purpose
Host machinery			
1897	Swbeg	CCGTCACAGGTATTTATTCGGCG	Testing for recombination
2420	MFD	CGCCAGGGTTTTCCCAGTCAC	
Mismatched Covalent Circles			
2391	SeqattI1	CACAGGAAACAGCTATGACC	Amplification of the region of the cointegrate including the mismatches.
2393	SeqNar	GCTTAATGAATTACAACAGTACTG C	
Double cleavage			
1078	MV143	CCTCTTACGTGCCGATCAACGTCTC	Verification of the constructs in pSW plasmids
1962	DapA-R	GTGGTGCCAACAGAAACGATCGC	

571	lacI-F	CATTAATGCAGCTGGCACGA	Sequencing
2263	5- Eco-FRT-Eco	AATTCGAAGTTCCTATTCCGAAGTTCCTATTCTCTAGAAAGTATAGGAACTTCG	Insertion of a FRT site in pSW plasmids
2264	3- Eco-FRT-Eco	AATTCGAAGTTCCTATACTTTCTAGAGAATAGGAACTTCGGAATAGGAACTTCG	
1897	Swbeg	CCGTCACAGGTATTTATTCGGCG	Verification of the monomeric/dimeric insertion of pSW plasmids in the chromosome
1898	Swend	CCTCACTAAAGGGAACAAAAGCTG	
1319	insertion lac-a	TTCAACTAGTGCGTGTGGAATGTGACGATCTTCGCGTCACC	
1698	inv1_attL6	AATAATGATTTTATTTTGACTGATAGTGACCTGTTCGTTGC	
1897	Swbeg	CCGTCACAGGTATTTATTCGGCG	Verification of the inversion of the region between inverted *attI* sites (double cleavage)
2933	Directionel Rec DAP	CATACTCGTATGTTGTGTGG	
930	J23100-R	CACTGTACCTAGGACTGAGCTAGCC	
1897	Swbeg	CCGTCACAGGTATTTATTCGGCG	Verification of recombination between direct *attI* sites
1962	DapA-R	GTGGTGCCAACAGAAACGATCGC	
Cleavage site: deep sequencing			
2915	mutatti WT F	GCGCCTCGAGGTTCGGTTAATGTTATGGAGCAGCAACGATGTTACGCAGCAGGGCAGTCG	Amplification of *attI* sites with random bases to build the library of $attI_N$
2916	mutatti WT R A1	GCGCCTGCAGTCCCGTGGCGTAACTTTGNTTTAGGGCGACTGCCCTGCTGCGTAACATCG	
2917	mutatti WT R A2	GCGCCTGCAGTCCCGTGGCGTAACTTTGTNTTAGGGCGACTGCCCTGCTGCGTAACATCG	
2918	mutatti WT R A3	GCGCCTGCAGTCCCGTGGCGTAACTTTGTTNTAGGGCGACTGCCCTGCTGCGTAACATCG	
2919	mutatti WT R A4	GCGCCTGCAGTCCCGTGGCGTAACTTTGTTTNAGGGCGACTGCCCTGCTGCGTAACATCG	
2920	mutatti WT R C1	GCGCCTGCAGTCCCGTGGCGTAACTTTNTTTTAGGGCGACTGCCCTGCTGCGTAACATCG	
2921	mutatti WT R C2	GCGCCTGCAGTCCCGTGGCGTAACTTTGTTTTANGGCGACTGCCCTGCTGCGTAACATCG	
2922	mutatti WT R C3	GCGCCTGCAGTCCCGTGGCGTAACTTTGTTTTAGNGCGACTGCCCTGCTGCGTAACATCG	
2923	mutatti WT R C4	GCGCCTGCAGTCCCGTGGCGTAACTTTGTTTTAGGNCGACTGCCCTGCTGCGTAACATCG	
2924	mutatti WT R Control (G)	GCGCCTGCAGTCCCGTGGCGTAANTTTGTTTTAGGGCGACTGCCCTGCTGCGTAACATCG	
2925	mutatti WT T	GCGCCTGCAGTCCCGTGGCGTAACTTTGTTTTNGGGCGACTGCCCTGCTGCGTAACATCG	
3449	attIN's right	TTTGATTTGAGCTCCACCGC	Amplification of the *attI* sites from the library and the
1236	MV202	TCAACGGGAATCCTGCTCTG	

738	MV84	CGACCATATGAAGAGGATGCCGCT AGGACC	cointegrates.
1896	MRV	AGCGGATAACAATTTCACACAGGA	
**In vitro**			
3885	attI WT top	TTTGATGTTATGGAGCAGCAACGA TGTTACGCAGCAGGGCAGTCGCCC TAAAACAAAGTTAGGCATCA	Construction of *attI1* substrates for *in vitro* experiments.
3886	attI WT bot	TGATGCCTAACTTTGTTTTAGGGCG ACTGCCCTGCTGCGTAACATCGTTG CTGCTCCATAACATCAAA	
3887	attI TTT top	TTTGATGTTATGGAGCAGCAACGA TGTTACGCAGCAGGGCAGTCGCCC TAAAACAAATTTAGGCATCA	
3888	attI TTT bot	TGATGCCTAAATTTGTTTTAGGGCG ACTGCCCTGCTGCGTAACATCGTTG CTGCTCCATAACATCAAA	
3889	attI L:AAATC R:WT top	TTTGATGTTATGGAGCAGCAACGA TGTTACGCAGCAGGGCAGTCGCCC TAAATCAAAGTTAGGCATCA	
Influence of T_{23} in the* attC *x* attI *reaction			
3849	$attC_{aadA7}$-Fw	AATTCGTCTAACAATTCATTCAAGC CGACGCCGCTTCGCGGCGCGGCTT AATTCAAGCGTTAGACG	Construction of the wild type *aadA7* site
3850	$attC_{aadA7}$-Rev	GATCCGTCTAACGCTTGAATTAAGC CGCGCCGCGAAGCGGCGTCGGCTT GAATGAATTGTTAGACG	
3571	$attC_{aadA7}$-ΔT_{23} Fw	GATCCGTCTAACGCTTGAATTAAGC CGCGCCGCGAAGCGGCGCGGCTTG AATGAATTGTTAGACG	Construction of the ΔT23 *aadA7* site
3572	$attC_{aadA7}$-ΔT_{23} Rev	AATTCGTCTAACAATTCATTCAAGC CGCGCCGCTTCGCGGCGCGGCTTA ATTCAAGCGTTAGACG	
1897	Swbeg	CCGTCACAGGTATTTATTCGGCG	Determination of the orientation and recombination box.
1898	Swend	CCTCACTAAAGGGAACAAAAGCTG	
1895	MFD	CGCCAGGGTTTTCCCAGTCAC	
571	lacI-F	CATTAATGCAGCTGGCACGA	
3883	RpLp	GGATCCGTCTAACGCTTG	
3884	RppLpp	GAATTCGTCTAACAATTC	

Suplementary references

1. Demarre, G. et al. A new family of mobilizable suicide plasmids based on the broad host range R388 plasmid (IncW) or RP4 plasmid (IncPa) conjugative machineries and their cognate *E.coli* host strains. *Research in Microbiology* **156**, 245-55 (2005).
2. Loot, C., Ducos-Galand, M., Escudero, J.A., Bouvier, M. & Mazel, D. Replicative resolution of integron cassette insertion. *Nucleic Acids Res* **40**, 8361-70 (2012).
3. Studier, F.W., Rosenberg, A.H., Dunn, J.J. & Dubendorff, J.W. Use of T7 RNA polymerase to direct expression of cloned genes. *Methods Enzymol* **185**, 60-89 (1990).

4. Biskri, L., Bouvier, M., Guerout, A.M., Boisnard, S. & Mazel, D. Comparative Study of Class 1 Integron and *Vibrio cholerae* Superintegron Integrase Activities. *J Bacteriol* **187**, 1740-50 (2005).
5. Demarre, G., Frumerie, C., Gopaul, D.N. & Mazel, D. Identification of key structural determinants of the IntI1 integron integrase that influence *attC* x *attI1* recombination efficiency. *Nucleic Acids Res* **35**, 6475-89 (2007).
